# Surgical management and postoperative outcomes of orbital cavernous malformations: A systematic literature review by the EANS skull base section

**DOI:** 10.1016/j.bas.2025.104302

**Published:** 2025-06-22

**Authors:** Edoardo Agosti, Vittorio Ricciuti, Giorgio Mantovani, Giorgia De Rosa, Pier Paolo Panciani, Marco Maria Fontanella, Cesare Zoia, EANS Skull Base Section, Moncef Berhouma, Michaël Bruneau, Luigi Maria Cavallo, Jan Frederick Cornelius, Sebastien Froelich, Emmanuel Jouanneau, Diego Mazzatenta, Torstein R. Meling, Mahmoud Messerer, Dimitris Paraskevopoulos, Pierre-Hugues Roche, HenryWS. Schroeder, Marcos Tatagiba, Idoya Zazpe, Roy Thomas Daniel

**Affiliations:** g Department of Neurosurgery, University Hospital of Dijon Bourgogne, Dijon, France; h Department of Neurosurgery, Universitair Ziekenhuis Brussel (UZ Brussel), Brussels, Belgium; i Neuroprotection and Neuromodulation (NEUR) Research Group, Center for Neurosciences (C4N), Vrije Universiteit Brussel (VUB), Brussels, Belgium; j Department of Neurosciences, Reproductive and Odontostomatological Sciences, Division of Neurosurgery, University of Naples Federico II, Naples, Italy; k Department of Neurosurgery, Medical Faculty & University Hospital, Heinrich Heine University Düsseldorf, Düsseldorf, Germany; l Department of Neurosurgery, Hopital Lariboisiere, Paris, France; m Department of Skull Base and Pituitary Surgery, Pituitary Tumors Centers of Excellence (PTCOE) of Lyon, Hôpital Pierre Wertheimer, Hospices Civils de Lyon, Lyon, France; n Department of Biomedical and Neuromotor Sciences (DIBINEM), University of Bologna, Bologna, Italy; o Programma Neurochirurgia Ipofisi - Pituitary Unit, IRCCS Istituto delle Scienze Neurologiche di Bologna, Bologna, Italy; p Neurosurgery, Copenhagen University Hospital, Rigshospitalet, Denmark; q Department of Neurosurgery, University Hospital of Lausanne and University of Lausanne, Lausanne, Switzerland; r Department of Neurosurgery, Barts Health NHS Trust, The Royal London Hospital, London, United Kingdom; s Department of Neurosurgery, Hôpital Nord, APHM, Marseille, France; t Department of Neurosurgery, University Medicine Greifswald, Greifswald, Germany; u Department of Neurosurgery and Neurotechnology, Eberhard Karls University, Tübingen, Germany; v Department of Neurosurgery, University Hospital of Navarre, Pamplona, Spain; a Department of Medical and Surgical Specialties, Division of Neurosurgery, Radiological Sciences and Public Health, University of Brescia, Piazza Spedali Civili 1, 25123 Brescia, Italy; b School of Medicine and Surgery, University of Milano-Bicocca, Monza, Italy; c Neurosurgery, Fondazione IRCCS San Gerardo dei Tintori, Monza, Italy; d Department of Translational Medicine, University of Ferrara, Ferrara, Italy; e Neurosurgery Department, S. Anna University Hospital of Ferrara, Ferrara, Italy; f Neurosurgical Unit, Ospedale Moriggia Pelascini, Gravedona e Uniti, Italy

**Keywords:** Cavernous malformations, Orbit, Systematic review, Surgical approaches, Outcomes

## Abstract

**Introduction:**

Orbital cavernous malformations (OCMs) are benign vascular lesions frequently associated with progressive proptosis and visual disturbances due to their slow growth and compression of adjacent structures. Multiple surgical approaches have been developed for their treatment, including microsurgical transfacial-transorbital approaches (MTTAs), cranio-orbital approaches (MCOAs), orbitotomies (MOs), endoscopic endonasal approaches (EEAs), and endoscopic transorbital approaches (ETOAs). However, the optimal approach remains a topic of debate.

**Research objective:**

This systematic review aims to compare the resection rates, postoperative complications, and clinical outcomes across various surgical approaches for OCM management.

**Methods:**

A comprehensive literature search was performed in PubMed, Embase, and the Cochrane Library according to PRISMA guidelines. Studies reporting surgical treatment of OCMs with clinical outcome data were included. Study quality was assessed using the Newcastle-Ottawa Scale. Statistical analyses were conducted using chi-square and Mann-Whitney U tests.

**Results and conclusions:**

Of 239 screened studies, 94 met inclusion criteria, comprising 1007 patients (mean age 43.9 years; 58.5 % female). Proptosis (63.2 %) and visual impairment (48.1 %) were the most common symptoms. Most lesions were intraconal (80 %) and laterally positioned (42.8 %). EEAs were the most commonly used approach (40.1 %), followed by MOs (25.7 %) and MTTAs (21.6 %). Gross total resection was achieved in 93.7 % of cases. Complications were infrequent: visual acuity worsening (3.9 %), diplopia (2.4 %), and enophthalmos (1.7 %). Functional outcomes improved significantly, particularly visual acuity (65.1 %) and proptosis (61.6 %). EEAs provide high resection rates with minimal morbidity, especially for medial OCMs. ETOAs represent a promising, minimally invasive option for laterally located lesions.

## Highlights

• Endoscopic Endonasal Approaches (EEAs) Lead in Use and Outcomes.
• Emerging Role of Endoscopic Transorbital Approaches (ETOAs).
• Minimally Invasive Techniques Show Lower Complication Rates.
• Functional Outcomes Significantly Improved Post-Surgery.
• Surgeon Experience and Institutional Preference Still Drive Approach Selection.

## 1 Introduction

Cavernous malformations (CMs) are vascular anomalies characterized histopathologically by dilated vascular channels containing blood products at different stages of degradation. The walls of these caverns are composed of a delicate fibrous adventitia and a single layer of endothelial cells with incomplete or dysfunctional tight junctions, leading to increased vascular permeability and potential hemorrhage into the surrounding parenchyma (Agosti et al., 2019; Fontanella et al., 2021).

Among CMs, orbital cavernous malformations (OCMs) represent a distinct subset and are the most common benign intraconal, intraorbital vascular tumors in adults (Ansari and Mafee, 2005; Bleier et al., 2016a; Castelnuovo et al., 2015). Orbital cavernous malformations account for approximately 4.3 % of orbital neoplasms and 9–13 % of all intracranial CMs. These lesions typically present between the third and fifth decades of life and are usually unilateral (Ansari and Mafee, 2005; Agosti et al., 2021a; Boari et al., 2011). Their slow growth and location within the orbit commonly lead to progressive, painless axial proptosis, which is the most frequent clinical sign. Other symptoms include visual disturbances such as reduced visual acuity, visual field defects, and dyschromatopsia due to optic nerve compression. Additionally, diplopia and extraocular motility restrictions may arise from the involvement of adjacent orbital structures (Bleier et al., 2016a; Alexander et al., 2022; Clarós et al., 2019).

Although OCMs are the most common benign vascular tumors of the orbit in adults, they remain a rare clinical entity overall. Precise incidence data are scarce, but available literature suggests an estimated occurrence of approximately 0.1–0.6 cases per 100,000 individuals annually, a figure that likely includes both symptomatic and asymptomatic lesions discovered incidentally during imaging for unrelated conditions (Fontanella et al., 2021; Castelnuovo et al., 2015; Clarós et al., 2019). This low incidence has contributed to the limited availability of large-scale, prospective studies, resulting in a fragmented evidence base for management strategies. Despite their rarity, OCMs frequently prompt surgical intervention due to progressive symptoms and their management presents significant anatomical and technical challenges. The growing number of surgical approaches and evolving minimally invasive techniques further underscores the need for a systematic synthesis of the literature to inform best practices and optimize patient outcomes (Agosti et al., 2019; Ansari and Mafee, 2005).

Surgical removal of symptomatic OCMs poses a significant challenge due to the tendency of these lesions to adhere to surrounding tissues, the risk of intraoperative bleeding, the limited working space within the orbital apex, and the potential for nerve injury (Millesi et al., 2021; Schick et al., 2003a). The choice of surgical approach depends on several factors, including lesion size, location, and relationship to the optic nerve, but also surgeon's preference and familiarity with approach. Traditionally, microsurgical transfacial-transorbital approaches (MTTAs), microsurgical cranio-orbital approaches (MCOAs) and microsurgical orbitotomies (MOs) have been the standard techniques for OCM removal. However, these approaches may be associated with significant morbidity, including cosmetic concerns and post-operative complications related to extensive tissue dissection and reconstruction (Millesi et al., 2021; Alexander et al., 2022).

In recent years, endoscopic techniques have gained increasing attention for the management of orbital pathologies (Locatelli et al., 2024; Agosti et al., 2024; Roccuzzo et al., 2024). Endoscopic endonasal approaches (EEAs) and endoscopic transorbital approaches (ETOAs) offer minimally invasive alternatives to traditional open approaches, potentially reducing surgical morbidity while maintaining efficacy (Chen et al., 2010; Bleier et al., 2016a; Clarós et al., 2019). EEAs provide a direct route to medially and inferiorly located OCMs, allowing access to the orbital apex without external incisions, brain retraction, or extensive bony removal. Initially employed for optic nerve decompression and drainage of orbital abscesses, the indications for EEAs have expanded to include tumor biopsy, debulking, and radical resection (Limawararut et al., 2006; Harris, 2010; Bleier et al., 2016a). Conversely, ETOAs, which access the orbit through small lateral or superior incisions, have emerged as a complementary technique, particularly for laterally positioned lesions. Some studies suggest that a combined endonasal and transorbital endoscopic approach may optimize surgical exposure and outcomes in select cases (Agosti et al., 2021b; Dallan et al., 2015a; Di Somma et al., 2018).

The relevance of OCMs extends beyond their rarity. These lesions, while benign, are often located in anatomically complex and functionally critical areas of the orbit, where even slow growth can lead to debilitating symptoms such as vision loss or disfigurement. As the use of high-resolution imaging has increased, incidental detection of these lesions has become more common, prompting the need for clear clinical guidelines on whether to observe or operate. Furthermore, the emergence of less invasive surgical techniques has generated ongoing debate regarding the optimal approach for different lesion locations. Despite these advances, comparative data across treatment modalities remain limited. A systematic review of the literature is warranted to assess the efficacy and safety of these different surgical strategies in the management of OCMs. Thus, this systematic review aims to compare MTTAs, MCOAs, MOs, EEAs, and ETOAs for OCM treatment, to clarify surgical outcomes and complications, inform clinical decision-making, and identify areas requiring further research in the management of OCMs.

## 2 Materials and methods

### 2.1 Literature search

A comprehensive and systematic literature search was conducted using PubMed, Embase, and the Cochrane Library, covering studies published up to December 2024. The search strategy was developed in accordance with the Preferred Reporting Items for Systematic Reviews and Meta-Analyses (PRISMA) guidelines (Page et al., 2021). Medical Subject Headings (MeSH) terms and free-text keywords related to OCMs and to surgical interventions were employed, including “orbital cavernous malformation," “orbital cavernous hemangioma," “surgical treatment," “microsurgical cranio-orbital approach," “microsurgical orbitotomy," “endoscopic endonasal approach," and “endoscopic transorbital approach." Boolean operators (AND, OR) were used to refine the search strategy and maximize the inclusion of relevant studies. The following search string was used: (("orbit cavernous malformation" OR “orbit cavernous hemangioma" OR “orbit vascular tumor") AND ("surgical treatment")). Additional records were identified through backward and forward citation tracking of selected articles. Only studies published in English and appearing in peer-reviewed journals were considered. Grey literature, including conference abstracts and unpublished manuscripts, was excluded to maintain a high standard of evidence reliability.

### 2.2 Study selection

After retrieving the initial pool of studies, duplicate records were removed. The remaining articles underwent a two-step screening process. In the first phase, four independent reviewers screened the titles and abstracts to exclude irrelevant studies. The second phase involved a full-text review of the remaining articles to determine their eligibility based on predefined inclusion and exclusion criteria. Studies were included if they reported on patients diagnosed with OCMs who underwent surgical treatment and provided clinical or surgical outcome data. Eligible studies included retrospective and prospective cohort studies, case reports and case series, and comparative studies analyzing different surgical approaches.

Exclusion criteria comprised studies focused exclusively on non-surgical management, those lacking sufficient outcome data, review articles, expert opinions, and studies involving pediatric populations if results were not analyzed separately. Disagreements between reviewers were resolved by discussion or consultation with a fifth independent investigator to ensure objectivity and consistency in study selection.

### 2.3 Data extraction

Data extraction was carried out independently by two authors using a standardized extraction form. Extracted data encompassed study characteristics (author, year, journal, country, study design, study period), patient demographics (sample size, mean age, sex distribution), clinical presentation (visual impairment, proptosis, diplopia, enophthalmos, extraocular muscle dysfunction, cranial nerve (CN) deficits), details on surgical approach (MTTAs, MCOAs, MOs, EEAs, and ETOAs), follow up period, details on postoperative surgical – including amount of resection (i.e. subtotal resection (STR), gross total resection (GTR), biopsy, optic canal (OC) decompression) and post-operative complications – and postoperative clinical outcomes.

### 2.4 Outcomes

Primary clinical outcomes included detailed ophthalmological assessments following surgery. These comprised changes in visual acuity (categorized as improved, unchanged, or worsened), presence or resolution of visual field deficits and color vision disturbances, evaluation of diplopia (new onset, persistent, or resolved), assessment of extraocular muscle function, and documentation of any new or worsening CN deficits affecting ocular motility or sensation. Primary surgical outcomes included the extent of tumor resection, classified as GTR, STR, biopsy only, or optic canal decompression. In addition, we evaluated the rate and type of postoperative complications, such as worsening visual function, diplopia, enophthalmos, CSF leak, infection, or hematoma formation.

### 2.5 Risk of bias assessment

**Fig. 1:**
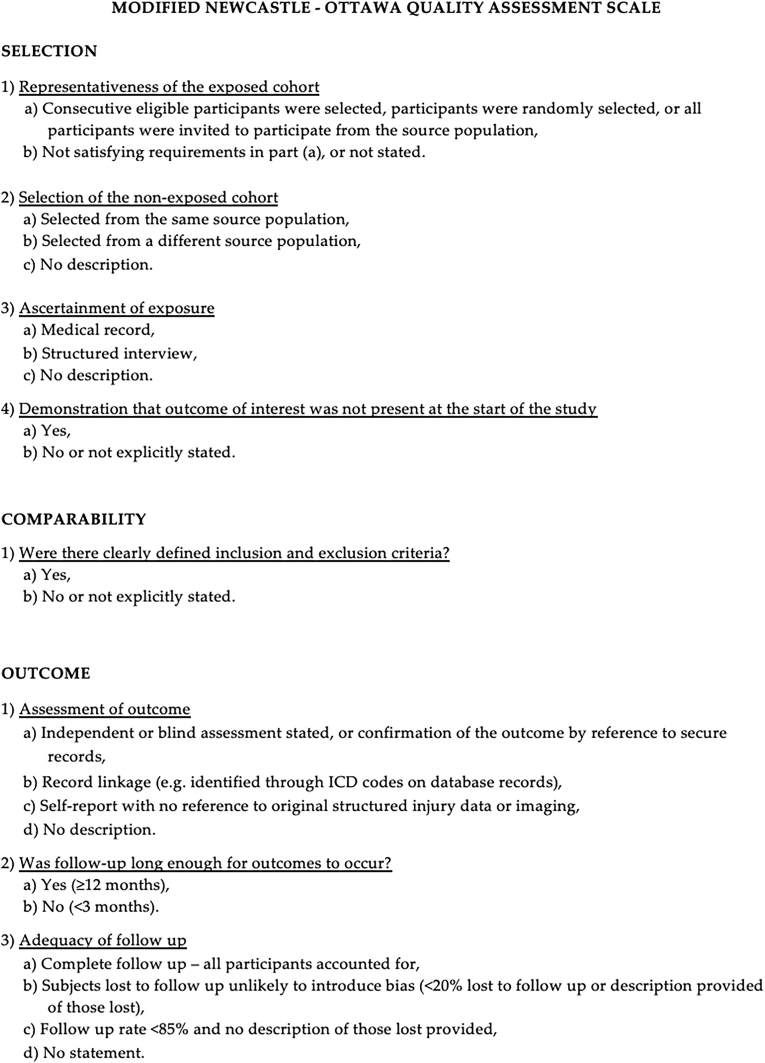
New Castel-Ottawa Scale flow chart.

The quality of the included observational studies was assessed using the Newcastle-Ottawa Scale (NOS), which evaluates studies based on selection, comparability, and outcome assessment domains (Fig. 1). (Stang, 2010) Each study was scored out of a maximum of 9 points. Studies scoring fewer than 7 points were considered low quality and excluded from the analysis. The remaining studies were included regardless of their score but were considered in subgroup analyses and narrative synthesis to interpret potential bias due to study quality. Detailed NOS scores for each study are provided in Supplementary material 1.

### 2.6 Statistical analysis

A descriptive statistical analysis of proportions was conducted to summarize the distribution of variables. Subsequently, the Chi-square goodness-of-fit test was performed to evaluate whether the observed frequencies significantly deviated from the expected distribution, with statistical significance defined as p < 0.05.

## 3 Results

### 3.1 Literature review

**Fig. 2:**
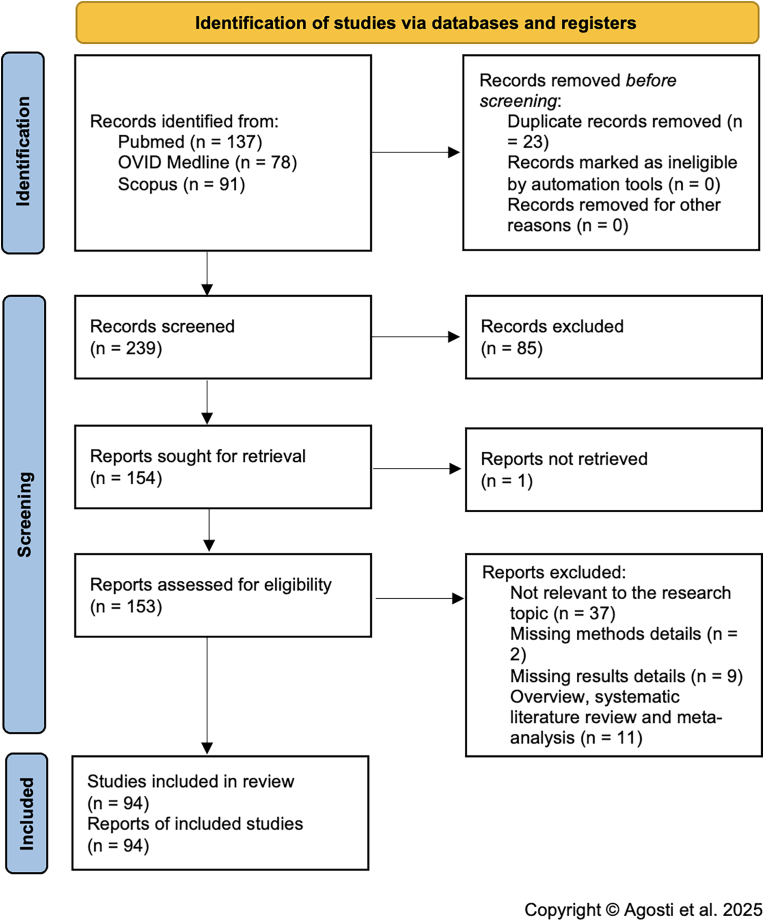
PRISMA flow chart.

A total of 239 papers were found after duplicates were eliminated. After titles and abstracts were examined, 154 papers were chosen for full-text evaluation. A total of 94 of the 153 papers that had their eligibility evaluated were included in the final assessment. The following standards were used to weed out the remaining 59 articles: 2 and 9 publications for the lack of methodological or results-related information respectively, 11 systematic literature reviews and meta-analyses, and 37 publications were not pertinent to the research topic. The PRISMA flowchart is summarized in Fig. 2. Prisma checklist is reported in Supplementary material 2. Table 1 includes details of anamnestic and clinical data of the studies included in the systematic review. Details of the radiological and surgical data from the studies that were part of the systematic review are included in Table 2. Details of clinical and surgical postoperative outcome data and follow-up duration of the studies that were part of the systematic review are detailed in Table 3.

**Table 1:** Anamnestic and clinical data of the included studies.

Author, year	Period	Sample size	Anamnestic data		Clinical presentation					Ophthalmological signs		
			Mean Age at Intervention	Females	Visual Impairment	Proptosis	Pain	Diplopia	Ocular movement deficit	Retinopathy	Optic Neuropathy	Corneal exposure Ocular movement deficit
Hobbs et al., 1961	1953	1	48	0	1	1	0	1	1	0	0	0
Silva and Symon, 1984	/	2	35.5	2	2	2	0	0	2	0	2	0
Dyer et al., 1985	1977-1984	3	44	0	3	3	0	0	0	/	/	/
Lyness et al., 1986	1984	1	23	1	0	1	0	1	0	0	0	0
Shields et al., 1987	/	1	42	1	1	1	0	0	0	0	0	0
Ohbayashi et al., 1988	1980	2 lesions, 1 patient	45	1	1	1	0	1	1	0	0	0
McNab et al., 1989	1968-1988	85	47.8	50	50	77	8	6	0	0	0	0
Leatherbarrow et al., 1989	1980-1988	3	50	1	2	3	0	1	0	0	0	0
Leib et al. British., 1993	1982	1	32	1	1	1	0	0	0	0	0	0
Missori et al., 1994	1994	25	46	0.68	20	0	4	0	0	0	0	0
Hassler et al., 1994	/	1	59	0	1	1	0	0	0	0	0	0
Herman et al., 1999	/	1	56	1	1	1	0	0	0	0	0	0
Hejazi et al., 1999	1988-1997	15	46.2	8	13	11	0	0	2	0	0	0
Maus et al., 1999	/	1	35	1	0	1	0	0	0	0	0	0
D'hermies et al., 2000	2000	1	42	1	1	1	0	0	1	0	1	1
Christensen et al., 2002	1999	1	31	1	1	1	0	0	1	0	1	1
Kim et al., 2002	/	1	45	1	1	1	0	0	0	0	0	0
Schick et al., 2003	1988-2002	37	51.2	25	19	27	8	0	6	0	0	0
Papalkar et al., 2005	/	1	55	1	1	1	1	0	0	0	0	0
Monin et al., 2005	/	1	28	0	1	0	0	0	0	0	0	0
Karaki et al., 2006	/	1	50	1	0	0	0	0	0	0	0	0
Hejazi et al., 2007	1988-2005	19	49.1	11	17	17	0	7	7	/	/	/
Chaddad Neto et al., 2007	/	1	39	1	0	1	0	0	0	0	0	0
Maheshwari et al., 2007	2002	1	4	1	1	1	0	0	0	1	0	0
Cheng et al., 2008	2001-2005	39	45.4	21	9	32	18	5	0	0	0	0
Yan et al., 2008	2005	1	54	0	0	1	0	0	0	0	0	0
Stamm et al., 2009	/	1	33	0	1	1	0	0	1	0	0	0
Tang Cho et al., 2010	2010	10	50	6	4	0	0	0	2	2	0	0
Yoshimura et al., 2010	2010	1	48	0	1	1	0	0	0	0	1	0
Cho et al., 2010	1998-2006	9	40.9	8	2	6	0	0	4	1	0	0
Gazioglu et al., 2011	/	1	50	0	1	0	0	0	0	0	0	0
Campbell et al., 2011	/	1	57	1	1	1	0	0	0	0	0	0
Boari et al., 2011	1999-2009	20	45	14	5	12	6	5	0	0	0	0
Locatelli et al., 2011	2012	1	59	1	1	1	0	0	0	0	0	0
Arora et al., 2011	/	1	40	1	1	1	0	0	1	0	0	0
Muscatello et al., 2012	2011	3	54	2	0	2	0	0	0	0	0	0
Yamamoto et al., 2012	/	1	61	0	0	1	1	1	1	0	0	0
Meena et al., 2012	/	1	29	0	0	1	0	0	0	0	0	0
Netuka et al., 2013	2010-2011	2	57.5	2	2	0	0	0	0	0	0	0
Wu et al., 2013	2008-2012	12	42.6	8	12	/	0	0	0	0	0	0
Aymard et al., 2013	2013	43	/	25	13	10	2	3	/	9	/	/
Murray et al., 2013	/	1	43	0	0	0	0	0	0	0	0	0
Healy et al., 2014	/	1	39	1	1	1	0	0	0	0	0	0
Chhabra et al., 2014	2007-2011	5	54.4	2	5	5	0	3	0	0	0	0
Yang et al., 2014	2002-2010	74	41.1	47	43	53	12	3	34	0	15	0
Dallan et al., 2015	/	1	/	1	1	1	0	0	0	0	1	0
Koch et al., 2015	2014	1	51	1	1	1	0	0	0	0	0	0
Ikonomidis et al., 2015	/	1	57	0	1	1	0	0	0	0	0	0
Kang et al., 2016	2016	1	59	1	1	1	0	0	0	0	1	0
Chen et al., 2016	2009-2016	9	43.5	2	11	0	0	0	0	0	11	0
Bleier et al., 2016	/	23	50.9	13	15	8	6	5	0	0	0	0
Xue et al., 2016	/	3	47.3	3	2	0	0	0	0	0	0	0
Wang et al., 2017	2014	9	49.1	6	5	9	0	0	0	0	3	0
Louisraj et al., 2017	2017	1	26	1	0	1	1	1	1	0	0	0
Bagheri et al., 2018	2001-2016	60	40	36	9	54	6	1	/	24	/	/
Bagheri et al., 2018	/	1	28	0	1	1	0	0	0	0	0	0
Golden et al., 2018	2018	10	41	/	0	1	0	0	0	0	0	/
Dallan et al., 2019	2008-2017	23	46.1	13	14	8	0	3	1	0	0	0
Marcellino et al., 2019	/	1	52	1	1	0	0	0	0	0	0	0
Castelnuovo et al., 2019	/	2	34	/	1	2	0	0	0	0	0	0
Claros et al., 2019	1997-2017	76	37.8	42	32	76	14	10	10	31	17	9
Hegde et al., 2019	/	1	65	1	0	1	1	1	0	0	0	0
Kim et al., 2019	2006-2017	18	44.8	14	5	16	1	0	6	0	0	0
Zoli et al., 2019	2019	1	47	0	0	1	1	1	1	1	1	1
An et al., 2020	2018	1	47	1	0	0	0	0	0	0	0	0
Quin et al., 2020	/	1	53	1	1	1	0	0	0	0	0	0
Rimmer et al., 2020	201-2018	7	/	/	3	0	0	1	0	0	0	0
May et al., 2020	/	1	62	0	0	1	0	0	0	0	0	0
Strianese et al., 2021	2005-2016	164	44.4	95	50	132	13	21	35	23	/	/
Fong Ng et al., 2021	/	1	64	1	1	0	0	0	0	0	0	0
De Feudis et al., 2021	/	1	42	1	0	1	1	0	0	0	0	0
Li et al., 2021	2016-2020	18	49.9	11	10	8	5	3	/	/	/	/
Almeida et al., 2021	2019	1	40	1	/	1	0	0	0	0	0	0
Millesi et al., 2021	2002-2019	35	50	22	11	15	0	4	0	0	0	0
Lao et al., 2021	/	1	37	0	1	1	0	0	0	0	1	0
Zoli et al., 2021	2002-2018	4	58	3	2	0	0	1	0	0	0	0
Austria et al., 2021	/	1	66	1	0	1	0	0	0	0	0	0
Li et al., 2021	/	1	68	1	1	0	0	0	0	0	0	0
de Melo Junior et al., 2021	2021	1	62	0	0	0	0	0	1	0	0	0
Park et al., 2022	/	1	43	0	1	1	0	0	0	0	1	0
Zhou et al., 2022	2018-2021	19	45.11	9	14	0	0	0	0	0	6	0
Essayed et al., 2022	/	1	47	1	0	1	1	0	0	0	0	0
Yang et al., 2022	2003-2020	35	43.3	25	35	0	0	0	0	0	0	0
Ayoub et al., 2022	2022	1	49	1	0	0	0	0	0	0	0	0
Almatrudi et al., 2023	/	1	1	0	0	1	0	0	1	0	0	1
Lai et al., 2023	/	1	14	1	0	0	0	0	1	0	0	0
Leocata et al., 2023	/	1	45	1	1	0	0	0	0	0	0	0
Jaxa-Kwiatkowski et al., 2023	/	1	35	/	1	1	0	0	0	0	0	0
Finisanti et al., 2023	/	1	20	1	0	1	1	0	0	0	0	0
Das et al., 2023	/	3	26.7	2	3	3	0	0	3	0	0	0
Dalfino et al., 2023	2023	1	55	1	1	1	0	0	0	0	0	0
Gulsuna et al., 2024	2018-2023	13	35.1	8	7	2	2	5	1	0	0	0
Kushwaha et al., 2024	/	1	48	1	1	1	0	1	1	0	0	0
Abdulla et al., 2024	2024	1	1	0	1	0	0	0	0	0	0	0

**Table 2:** Radiological and surgical data of the included studies.

Author, year	Location			Position				Size (cm)	Surgical Treatment				
	Intraconal	Extraconal	Optic Canal	Medial	Lateral	Superior	Inferior		EEA	MTTA	MCOA	MO	ETOA
Hobbs et al., 1961	NA	NA	NA	1	0	0	0	2.8x2.3x1.4	0	1	0	0	0
Costa e Silva et al., 1984	0	0	2	0	0	0	0	0.45x0.35	0	0	2	0	0
Dyer et al., 1985	NA	NA	0	0	2	0	0	3 × 3 × 2	0	0	3	0	0
Lyness et al., 1986	1	0	0	0	1	0	0	5x4x4	0	0	0	1	0
Shields et al., 1987	1	0	0	0	1	0	0	/	0	0	0	1	0
Ohbayashi et al., 1988	0	2	0	0	2	0	0	2.8x1.8	0	0	1	1	
McNab et al., 1989	NA	NA	2	16	65	29	26	/	0	10	4	71	0
Leatherbarrow et al., 1989	3	0	0	3	0	0	0	/	0	3	0	0	0
Leib et al. British., 1993	1	0	0	1	0	0	0	2.3x1.7x1.4	0	0	0	1	0
Missori et al., 1994	0	0	0	0	0	0	0	/	0	0	0	0	0
Hassler et al., 1994	1	0	0	0	0	0	1	4x2	0	1	0	0	0
Herman et al., 1999	1	0	0	1	0	0	1	1.1x0.8x1.2	1	0	0	0	0
Hejazi et al., 1999	13	2	0	7	3	3	0	/	0	4	8	3	0
Maus et al., 1999	1	0	0	0	1	0	0	/	0	0	1	0	0
D'hermies et al., 2000	1	0	0	1	1	1	0	5x4x3	0	0	1	0	0
Christensen et al., 2002	1	0	0	1	1	1	0	5x4x3	0	0	1	0	0
Kim et al., 2002	1	0	0	0	0	0	1	2.3x3x3x7	0	0	0	1	0
Schick et al., 2003	31	5	1	13	15	7	13	/	1	9	17	10	0
Papalkar et al., 2005	1	0	0	0	0	1	0	2x1.5x1	0	0	1	0	0
Monin et al., 2005	0	0	1	0	0	0	0	1.4x0.5	1	0	0	0	0
Karaki et al., 2006	1	0	0	1	0	0	0	/	1	0	0	0	0
Hejazi et al., 2007	19	0	0	4	3	5	7	/	0	7	9	3	0
Chaddad Neto et al., 2007	/	/	/	1	0	1	0	/	0	0	1	0	0
Maheshwari et al., 2007	0	1	0	1	0	0	0	2.7x1.4x1.8	0	1	0	0	0
Cheng et al., 2008	39	0	0	4	35	9	30	/	0	39	0	0	0
Yan et al., 2008	0	1	0	0	0	0	1	1.7x2.2	0	1	0	0	0
Stamm et al., 2009	1	0	0	1	0	0	0	/	1	0	0	0	0
Tang Cho et al., 2010	/	0	0	0	0	/	/	/	0	0	0	0	10
Yoshimura et al., 2010	1	0	0	1	0	0	1	/	1	0	0	0	0
Cho et al., 2010	5	4	0	3	/	/	1	2.3x1.8	0	9	0	0	0
Gazioglu et al., 2011	1	0	0	1	0	0	0	/	1	0	0	0	0
Campbell et al., 2011	/	/	0	0	0	0	1	2.5x1.9x1x7	1	1	0	0	0
Boari et al., 2011	20	0	0	7	6	0	5	/	0	0	12	8	0
Locatelli et al., 2011	1	0	0	1	0	0	1	1.1x0.8x1.2	1	0	0	0	0
Arora et al., 2011	1	0	0	0	1	0	0	1.6x1	0	0	0	1	0
Muscatello et al., 2012	2	1	0	2	0	0	1	/	2	1	0	0	0
Yamamoto et al., 2012	1	0	0	0	1	0	1	/	0	0	1	0	0
Meena et al., 2012	1	0	0	0	1	0	1	2x1.5x1.5	0	0	0	1	0
Netuka et al., 2013	1	1	1	2	0	0	0	/	2	0	0	0	0
Wu et al., 2013	/	/	2	9	0	0	1	2x1.2	12	0	0	0	0
Aymard et al., 2013	15	8	/	0	0	/	/	/	0	/	0	/	0
Murray et al., 2013	1	0	0	1	0	0	0	1.6x1.8x1.5	1	0	0	0	0
Healy et al., 2014	1	0	0	1	0	0	1	1.3x1.3x1.3	1	0	0	0	0
Chhabra et al., 2014	/	/	/	5	0	0	0	1.68	5	0	0	0	0
Yang et al., 2014	74	0	0	7	40	14	13	/	0	69	0	5	0
Dallan et al., 2015	1	0	0	1	0	1	0	/	0	0	1	0	0
Koch et al., 2015	1	0	0	1	0	1	0	/	0	0	1	0	0
Ikonomidis et al., 2015	1	0	0	0	0	0	1	1.7x2.1	1	0	0	0	0
Kang et al., 2016	1	0	0	1	0	0	1	/	1	0	0	0	0
Chen et al., 2016	0	0	11	0	0	0	0	0-5x0.4x0.3	11	0	0	0	0
Bleier et al., 2016	14	3	5	/	/	/	/	1.57x1.15x1.09	23	0	0	0	0
Xue et al., 2016	3	0	0	1	1	0	1	/	0	1	2	0	0
Wang et al., 2017	6	3	0	9	0	2	7	1.9x1.6x1.3	9	0	0	0	0
Louisraj et al., 2017	1	0	0	0	1	0	1	/	0	0	1	0	0
Bagheri et al., 2018	58	2	/	9	49	/	/	/	0	10	0	50	0
Bagheri et al., 2018	0	1	0	0	0	0	1	3.5x2.5x2	0	0	0	1	0
Golden et al., 2018	/	/	0	0	0	0	/	/	0	0	10	0	0
Dallan et al., 2019	16	7	0	23	0	/	/	1.9x1.5x1.4	23	0	0	0	0
Marcellino et al., 2019	0	1	0	1	0	0	0	/	1	0	0	0	0
Castelnuovo et al., 2019	2	2	0	2	0	0	0	1.8x1.14	2	0	0	0	0
Claros et al., 2019	72	4	0	6	66	0	0	/	0	4	0	72	0
Hegde et al., 2019	1	0	0	0	1	0	0	2.7x1.4x1.4	0	0	0	1	0
Kim et al., 2019	18	0	0	7	11	6	12	/	0	0	0	8	0
Zoli et al., 2019	1	1	1	1	1	1	1	1	1	1	1	1	1
An et al., 2020	1	0	0	1	0	0	1	/	1	0	0	0	0
Quin et al., 2020	0	1	1	0	1	1	1	/	0	0	1	0	0
Rimmer et al., 2020	4	2	0	/	/	/	/	1.9x1.4x1.7	7	7	0	0	0
May et al., 2020	1	0	0	1	0	0	0	/	0	1	0	0	0
Strianese et al., 2021	130	34	40	49	100	58	52	/	0	0	130	0	0
Fong Ng et al., 2021	1	0	1	0	1	0	0	1x1x1	0	0	0	0	1
De Feudis et al., 2021	1	0	0	1	0	1	0	/	0	1	0	0	0
Li et al., 2021	12	6	/	16	1	4	12	2x1.6x1x6	16	2	0	0	0
Almeida et al., 2021	1	0	0	1	0	0	1	/	0	1	0	0	0
Millesi et al., 2021	31	4	0	6	13	7	9	2.1	0	13	14	8	0
Lao et al., 2021	1	0	0	1	0	0	1	2.6x1.6x1.9	1	0	0	0	0
Zoli et al., 2021	4	0	0	3	1	2	2	/	3	0	0	0	1
Austria et al., 2021	0	1	0	0	0	0	1	/	0	0	0	1	0
Li et al., 2021	0	1	0	1	0	0	0	/	1	0	0	0	0
de Melo Junior et al., 2021	0	1	0	0	0	1	0	2.3x1.3x2	0	1	0	0	0
Park et al., 2022	1	0	0	1	0	0	0	2x1.5x1.7	1	0	0	0	0
Zhou et al., 2022	19	0	/	/	/	/	/	/	19	0	0	0	0
Essayed et al., 2022	1	0	0	1	0	0	0	/	0	0	1	0	0
Yang et al., 2022	35	0	0	26	0	0	9	/	22	0	13	0	0
Ayoub et al., 2022	1	0	0	1	0	0	1	/	1	0	0	0	0
Almatrudi et al., 2023	1	0	0	0	1	1	0	/	0	1	0	0	0
Lai et al., 2023	0	1	0	0	0	1	0	2.3x1.3x2	0	1	0	0	0
Leocata et al., 2023	1	0	0	1	0	0	0	/	1	0	0	0	0
Jaxa-Kwiatkowski et al., 2023	1	0	0	1	0	0	0	4.3x3.5x3.4	0	0	1	0	0
Finisanti et al., 2023	0	1	0	0	1	0	0	/	1	0	0	0	0
Das et al., 2023	3	0	0	2	1	0	0	/	0	0	1	2	0
Dalfino et al., 2023	1	0	0	1	0	1	0	/	0	0	1	0	0
Gulsuna et al., 2024	12	1	3	13	0	2	3	/	13	0	0	0	0
Kushwaha et al., 2024	1	0	0	1	0	0	0	2x2	0	1	0	0	0
Abdulla et al., 2024	0	1	1	0	1	1	1	/	0	0	1	0	0

**Table 3:** Postoperative clinical and surgical outcome data of the included studies.

Postoperative Clinical Outcomes					Postoperative Surgical Outcomes										FU (months)
					Resection				Complications						
Author, year	Visual Acuity Improvement	Ocular Movement Improvement	Proptosis Improvement	Diplopia Improvement	GTR	STR	Biopsy	OC decompression	Infections	Visual Acuity deficit appearance/worsening	Diplopia Appearance/Worsening	Enophthalmos	Ocular Movement deficit appearance/Worsening	Recurrence	
Hobbs, 1961	1	1	1	1	1	0	0	0	0	0	0	0	0	0	60
Costa e Silva et al., 1984	1	2	2	0	2	0	0	0	0	0	0	0	0	0	/
Dyer and Atkinson, 1985	1	0	0	0	3	0	0	0	0	1	1	1	0	0	6
Lyness and Williams, 1986	0	0	1	1	0	0	1	0	0	0	0	0	0	0	6
Shields et al., 1987	1	0	1	0	1	0	0	0	0	0	0	0	0	0	24
Ohbayashi et al., 1988	1	1	1	1	2	0	0	0	/	0	0	0	0	/	/
McNab and Wright, 1989	31	/	/	/	80	5	0	0	/	7	5	0	3	0	/
Leatherbarrow et al., 1989	1	0	2	1	1	0	2	0	0	0	0	0	1	1	17
Leib et al. British., 1993	1	0	1	0	1	0	0	0	0	0	0	0	0	0	/
Missori et al., 1994	/	/	8	/	0	4	0	3	0	0	0	0	0	0	/
Hassler et al., 1994	0	0	1	0	1	0	0	0	0	0	0	0	0	0	8
Herman et al., 1999	1	0	0	0	0	0	0	0	0	0	0	0	0	0	6
Hejazi et al., 1999	8	/	/	/	/	/	/	/	/	/	/	/	/	/	/
Maus et al., 1999	0	0	1	0	1	0	0	0	0	0	0	0	0	0	/
D'hermies et al., 2000	0	0	1	0	1	0	0	0	0	0	0	0	0	0	/
Christensen et al., 2002	0	0	1	0	1	0	0	0	0	0	0	0	0	0	24
Kim et al., 2002	1	0	1	0	1	0	1	0	0	0	0	0	0	0	6
Schick et al., 2003b	14	4	21	0	37	0	0	0	0	2	0	0	0	/	12.65
Papalkar et al., 2005	1	0	1	0	1	0	0	0	0	0	0	0	0	0	/
Monin et al., 2005	1	0	0	0	0	0	1	1	0	0	0	0	0	/	/
Karaki et al., 2006	0	0	0	0	1	0	0	0	0	0	0	0	0	0	/
Hejazi et al., 2007	14	/	/	7	/	/	/	/	/	0	0	0	0	/	37
Chaddad Neto et al., 2007	0	0	/	0	1	0	0	0	0	0	0	0	0	0	36
Maheshwari and Thool, 2007	0	0	1	0	1	0	0	0	0	0	0	0	0	1	11
Cheng et al., 2008	6	0	32	5	37	2	0	0	0	2	0	0	0	0	18
Yan et al., 2008	0	0	1	0	1	0	0	0	0	0	0	0	0	0	/
Stamm and Nogueira, 2009	1	/	/	/	1	0	0	0	0	0	0	0	0	0	/
Tang Cho et al., 2010	4	/	/	/	/	0	0	0	0	/	0	0	0	0	12
Yoshimura et al., 2010	1	0	1	0	1	0	1	/	1	0	0	0	0	0	8
Cho et al., 2010	2	4	9	0	9	0	0	0	0	0	0	0	0	0	29
Gazioglu et al., 2011	1	0	0	0	1	0	0	0	0	0	0	0	0	0	3
Campbell et al., 2011	1	0	0	0	1	0	0	0	0	0	0	0	0	0	3
Boari et al., 2011	4	0	12	2	20	0	0	0	0	1	0	0	0	0	47.15
Locatelli et al., 2011	1	0	1	0	1	0	0	0	0	0	0	0	0	0	5
Arora et al., 2011	/	/	/	/	1	0	0	0	/	/	/	/	/	/	/
Muscatello et al., 2013	1	0	0	0	3	0	0	0	0	0	0	0	0	0	/
Yamamoto et al., 2012	0	1	0	1	0	1	0	0	0	0	0	0	0	0	/
Meena et al., 2012	0	0	1	0	1	0	0	0	0	0	0	0	0	1	9
Netuka et al., 2013	2	0	0	0	2	0	0	0	0	0	0	0	0	0	21
Wu et al., 2013	12	0	0	0	12	0	0	0	0	0	0	0	0	0	11.8
Aymard et al., 2013	NA	NA	NA	NA	32	0	0	0	0	0	3	0	2	0	/
Murray et al., 2013	0	0	0	0	1	0	0	0	0	0	0	0	0	0	12
Healy et al., 2014	1	0	1	0	1	0	0	0	0	0	0	0	0	0	3
Chhabra et al., 2014	5	0	5	3	4	1	0	0	0	0	0	2	0	0	24
Yang and Yan, 2014	24	/	74	/	74	0	0	0	0	6	/	0	1	2	/
Dallan et al., 2015	1	0	1	0	1	0	0	0	0	0	0	0	0	0	12
Koch et al., 2015	1	0	1	0	1	0	0	0	0	0	0	0	0	0	15
Ikonomidis et al., 2015	1	0	0	0	1	0	0	0	0	0	0	1	0	0	3
Kang et al., 2016	1	0	1	0	1	0	0	0	0	0	0	0	0	0	12
Chen et al., 2016	11	0	0	0	11	0	0	11	0	0	0	0	0	0	9.9
Bleier et al., 2016b	12	/	8	3	17	2	2	2	/	0	6	5	/	/	/
Xue et al., 2016	3	0	0	0	3	0	0	0	0	0	0	0	0	0	6
Wang et al., 2017	3	0	9	0	9	0	0	0	0	2	0	6	0	0	4.5
Louisraj et al., 2017	0	1	0	1	0	1	0	0	0	0	0	0	0	0	11
Bagheri et al., 2018	/	/	/	/	60	0	0	0	0	0	4	0	4	0	61.4
Bagheri et al., 2018	1	0	1	0	1	0	0	0	0	0	0	0	0	0	3
Golden et al., 2018	0	0	0	0	0	/	/	/	0	0	0	0	/	/	/
Dallan et al., 2018	10	/	8	/	16	4	0	3	0	0	0	0	0	0	52.9
Marcellino et al., 2019	1	0	0	0	1	0	0	0	0	0	0	0	0	0	/
Castelnuovo et al., 2019	1	0	2	0	2	0	0	0	0	0	0	0	0	0	8
Claros et al., 2019	24	/	/	/	76	0	0	0	0	0	0	0	0	/	6
Hegde et al., 2019	1	0	1	1	1	0	0	0	0	0	0	0	0	0	12
Kim et al., 2019	4	/	16	/	/	/	/	/	0	0	0	0	1	0	11.2
Zoli et al., 2019	1	1	1	1	1	1	1	1	1	1	1	1	1	1	1
An et al., 2020	0	0	0	0	1	0	0	0	0	0	0	0	0	0	3
Quin et al., 2020	1	0	1	0	1	0	0	0	0	0	0	0	0	0	/
Rimmer et al., 2020	/	/	/	/	3	0	1	0	0	0	0	0	0	/	/
May et al., 2021	0	0	1	0	1	0	0	0	0	0	0	0	0	0	/
Strianese et al., 2021	42	25	130	16	164	0	0	0	0	2	5	0	0	0	77
Fong Ng et al., 2022	1	0	0	0	1	0	0	1	0	0	0	0	0	0	/
De Feudis et al., 2021	0	0	1	0	1	0	0	0	0	0	0	0	0	0	3
Li et al., 2021	/	/	/	/	14	1	0	3	0	4	0	0	0	0	6
Almeida et al., 2021	0	0	0	0	1	0	0	0	0	0	0	0	0	1	6
Millesi et al., 2021	6	0	15	2	35	0	0	0	0	0	1	0	0	/	/
Lao et al., 2021	/	/	/	/	1	0	0	0	0	/	/	/	/	/	/
Zoli et al., 2021	1	0	0	1	4	0	0	0	0	0	0	0	0	0	/
Austria et al., 2021	0	0	1	0	1	0	0	0	0	0	0	0	0	0	8
Li et al., 2021	1	0	0	0	1	0	0	0	0	0	0	0	0	0	3
de Melo Junior et al., 2021	0	0	0	0	1	0	0	0	0	1	0	0	0	0	1
Park et al., 2022	1	0	1	0	1	0	0	0	0	0	0	1	0	0	12
Zhou et al., 2022	14	0	0	0	19	0	0	0	0	0	0	0	0	0	6.71
Essayed and Al-Mefty, 2022	1	0	1	0	1	0	0	0	0	0	0	0	0	0	/
Yang et al., 2022	17	0	0	0	35	0	0	0	0	9	0	0	0	0	64.5
Ayoub et al., 2022	0	0	0	0	1	0	0	0	0	0	0	0	0	0	3
Almatrudi et al., 2023	0	1	1	0	/	/	/	0	0	0	0	0	0	0	/
Lai et al., 2023	0	0	0	0	1	0	0	0	0	0	0	0	0	0	36
Leocata et al., 2023	1	0	0	0	1	0	0	0	0	0	0	0	0	0	6
Jaxa-Kwiatkowski et al., 2023	1	1	1	0	1	0	0	0	0	0	0	0	0	0	3
Finisanti et al., 2023	0	0	1	0	1	0	0	0	0	0	0	0	0	0	3
Das et al., 2023	2	0	3	0	3	0	0	0	0	0	0	0	0	0	16
Dalfino et al., 2023	1	0	1	0	1	0	0	0	0	0	0	0	0	0	/
Gulsuna et al., 2024	4	1	2	4	13	0	0	0	0	1	0	0	0	0	21
Kushwaha et al., 2024	1	1	1	1	1	0	0	0	0	0	0	0	0	0	/
Abdulla et al., 2024	1	0	1	0	1	0	0	0	0	0	0	0	0	0	/

All included studies were assessed for methodological quality using the Newcastle-Ottawa Scale (NOS). A total of 94 studies underwent quality evaluation, and all were found to have a score equal to or greater than 7, reflecting an acceptable to high level of methodological rigor. Specifically, 41 studies (43.6 %) scored 7, 40 studies (42.6 %) scored 8, and 13 studies (13.8 %) scored 9. This distribution indicates a strong overall quality among the included studies. Although detailed NOS scoring is provided in Supplementary Material 1.

#### 3.1.1 Data analysis

##### 3.1.1.1 Anamnestic and clinical data

The study analyzed 1007 patients with a mean age of 43.9 years (range: 1–85). Females accounted for 58.5 % of the cohort, a significantly higher proportion compared to males (p = 0.04). Among clinical presentations, proptosis was the most frequent symptom (63.2 %), followed by visual impairment (48.1 %). Less common manifestations included ocular movement deficit, pain, and diplopia (p = 0.006). Regarding ophthalmological signs, retinopathy was the most prevalent finding (9.1 %), followed by optic neuropathy and corneal exposure due to ocular movement deficit (p < 0.0001) (Table 4).

**Table 4:** Summary of anamnestic and clinical data of the included studies.

**Total Patients N**			1007
**Anamnestic data**	Age means (range)		43.9 (1-85)
	Females %		58.5 (589/1007)
**Clinical data**	Clinical presentation %	Visual impairment	48.1 (485/1007)
		Proptosis	63.2 (637/1007)
		Pain	11.3 (114/1007)
		Diplopia	9.4 (95/1007)
		Ocular movement Deficit	12.5 (126/1007)
	Ophthalmological signs %	Retinopathy	9.1 (92/1007)
		Optic neuropathy	6.1 (62/1007)
		Corneal exposure ocular movement deficit	1.2 (13/1007)

##### 3.1.1.2 Radiological and surgical data

**Fig. 3:**
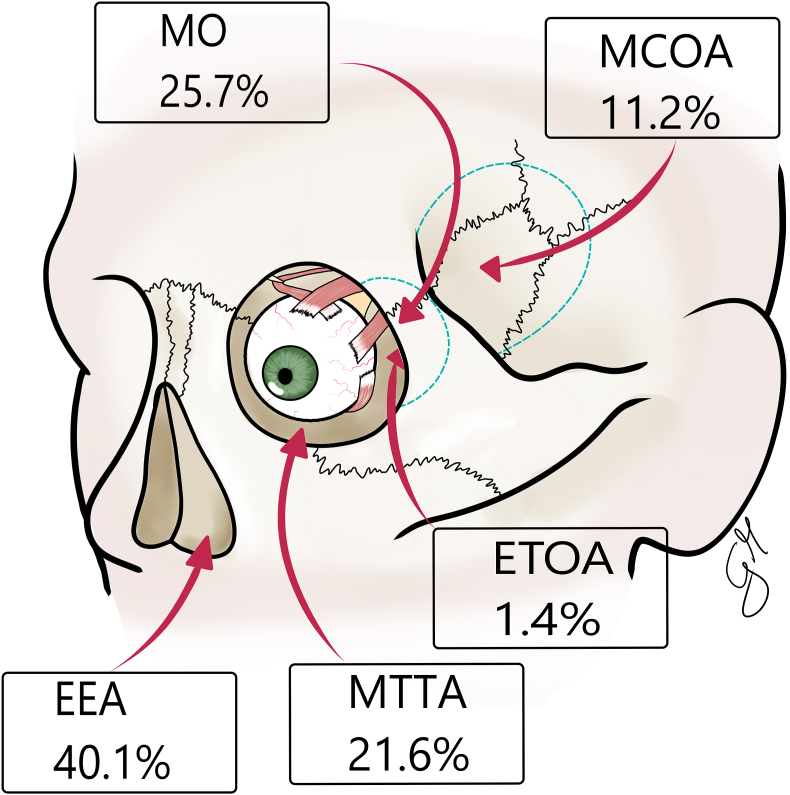
Schematic representation of the surgical approaches. MO: Microsurgical Orbitotomy, MCOA: Microsurgical Cranio-Orbital Approaches, MTTA: Microsurgical Transfacial-Transorbital approaches, EEA: Endoscopic Endonasal Approaches, ETOA: Endoscopic Transorbital Approaches.

Radiological analysis revealed a predominant intraconal localization (80 %), with significantly fewer cases in the extraconal space or optic canal (p < 0.001). Lesions were more frequently found in the lateral position (39 %), while medial, inferior, and superior locations were less common (p = 0.01). The selection of technique could vary significantly based on lesion location, underlying the importance of tailored surgical planning (Table 5). The EEAs were the most used approaches (40.1 %), followed by MOs and MTTAs (25.7 % and 21.6 % respectively) (Fig. 3).

**Table 5:** Summary of radiological and surgical data of the included studies.

**Radiological data**	Mean maximum diameter (cm) N (range)		3 (0.45–5)
	Location % (N/tot)	Intraconal	80 (701/876)
		Extraconal	11.8 (103/876)
		Optic canal	8.2 (72/876)
	Position % (N/tot)	Medial	25.8 (285/1104)
		Lateral	39 (431/1104)
		Superior	14.7 (162/1104)
		Inferior	20.5 (226/1104)
**Surgical approach % (N/tot)**		EEA	40.1 (393/979)
		MTTA	21.6 (211/979)
		MCOA	11.2 (110/979)
		MO	25.7 (251/979)
		ETOA	1.4 (14/979)

##### 3.1.1.3 Post-operative surgical and clinical outcomes

Postoperative outcomes varied significantly depending on the surgical approach (p < 0.001). Gross total resection was achieved in 93.7 % of cases, while STR, biopsy, and optic canal decompression were performed in a minority of patients (p < 0.001). Postoperative complications occurred at low rates, with visual acuity worsening (3.9 %), diplopia (2.4 %), enophthalmos (1.7 %), and ocular movement deficits (1.1 %) (p = 0.002). Clinical outcomes demonstrated significant improvements, with visual acuity improving in 65.1 %, ocular movement in 34.9 %, proptosis in 61.6 %, and diplopia in 56.8 % of cases (p = 0.03) (Table 6).

**Table 6:** Summary of postoperative clinical and surgical outcome data of the included studies.

**Postoperative surgical outcome % (N/tot)**	Resection	GTR	93.7 (821/876)
		STR	2.5 (22/876)
		Biopsy	0.9 (8/876)
		OC decompression	2.9 (25/876)
	Complications	Infection	0.1 (1/979)
		Visual acuity deficit appearance/worsening	3.9 (38/979)
		Diplopia appearance/worsening	2.4 (23/979)
		Enophthalmos	1.7 (17/979)
		Ocular movement deficit appearance/worsening	1.1 (11/979)
		Recurrence	0.7 (6/821)
**Postoperative clinical outcome % (N/tot)**		Visual acuity improvement	65.1 (316/485)
		Ocular movement improvement	34.9 (44/126)
		Proptosis improvement	61.6 (393/637)
		Diplopia improvement	56.8 (54/95)

## 4 Discussion

Our systematic review highlights the predominance of the EEAs over traditional techniques, with an emerging role in the last years of ETOAs. Gross total resection was achieved in majority of cases, with significant improvements in visual acuity, proptosis, and diplopia. The low complication rates reinforce the safety and efficacy of modern surgical strategies for orbital cavernous malformations.

### 4.1 Clinical presentations

The propensity for OCMs to cause visual disturbances is well-documented (Almeida et al., 2022; Lao et al., 2021; Austria et al., 2023). In our systematic review, nearly half of the patients reported visual impairment. The insidious growth pattern of OCMs often leads to delayed diagnoses. Clinical symptoms are frequently attributed to more common orbital pathologies, resulting in misdiagnoses. Our findings reveal that a significant proportion of patients experienced delayed recognition of their condition, emphasizing the need for heightened clinical suspicion, especially in cases presenting with unexplained proptosis or visual decline. A study by Yang et al. (2021) analyzed 35 patients with OCMs located under the optic nerve sheath in the orbital apex or common tendon ring. They reported a high rate of misdiagnosis (57.1 %), with many cases initially mistaken for optic neuritis. Additionally, 65.7 % of patients experienced acute or subacute visual acuity deterioration, primarily due to hemorrhagic events within the lesion. These acute presentations contrast with the more gradual symptom progression observed in our review, suggesting that lesion location, particularly in proximity to critical structures like the optic nerve, may precipitate more rapid clinical declines. A case reported by Lao et al. (2021) described a patient with an OCM in the infraorbital canal presenting with intense headaches and visual disturbances, initially misdiagnosed as an infraorbital nerve neurofibroma. This case highlights the diagnostic challenges posed by atypical clinical presentations.

The anatomical positioning of OCMs significantly influences their clinical manifestations (Zoia et al., 2024a; Fontanella et al., 2021; Castelnuovo et al., 2019). Our review indicates that 80 % of lesions are intraconal. This intraconal predominance often results in symptoms, such as proptosis and visual disturbances, due to mass effect on adjacent ocular structures. Conversely, extraconal lesions may present with less pronounced proptosis but can still cause significant visual impairment if they exert pressure on the optic apparatus.

While our systematic review focuses on surgical outcomes, it is important to acknowledge that not all OCMs require immediate intervention. The decision to pursue surgery versus conservative management is influenced by lesion size, growth rate, anatomical location, presence of symptoms (particularly visual impairment), and patient preference. Surveillance with periodic imaging is often recommended for small, asymptomatic, or incidentally discovered lesions. Surgical resection, which is indicated in the presence of progressive symptoms or radiological growth, remains the most frequently employed treatment modality, reported in 40–60 % of cases. Radiotherapy, while rarely used, may be considered in select situations involving unresectable lesions or high surgical risk.

### 4.2 Surgical treatment

Our systematic review highlights the predominance of the EEAs, followed by MOs, MCOAs, and ETOAs. This distribution underscores the evolving preference for less invasive techniques in the treatment of OCMs, aligning with recent literature emphasizing the benefits of minimally invasive approaches in reducing morbidity and improving patient outcomes.

Microsurgical cranio-orbital approaches, once considered the gold standard for deeply located OCMs, provide extensive exposure and direct visualization of the lesion but come with considerable drawbacks. Studies such as those by Yang et al. (2021) have reported increased operative times, risk of neurovascular injury, and prolonged recovery periods associated with these approaches. We reported a decreasing trend of MCOAs (10.9 %). Our data reflect this shift away from MTTAs and MCOAs, with their relatively low utilization suggesting that surgeons increasingly prioritize approaches that minimize surgical trauma while maintaining efficacy.

Microsurgical orbitotomies remain a widely used technique, particularly for lateral and extraconal lesions. They offer a balance between adequate exposure and minimized invasiveness compared to MTTAs and MCOAs, as confirmed by several studies (Aymard et al., 2013). However, complications such as ocular muscle damage, nerve injury, and postoperative diplopia remain concerns. The external incisions required for MOs also pose cosmetic considerations, leading to a preference for less invasive options when feasible.

Endoscopic endonasal approaches (EEAs) have emerged as the most frequently employed surgical technique, accounting for the largest proportion of cases in our review (Zoia et al., 2024b; Bleier et al., 2016a; An et al., 2020; Park et al., 2022). This approach offers a direct route to medial and inferomedial orbital lesions via the nasal corridor, avoiding external incisions and reducing the risk of cosmetic deformities. The literature supports this trend, with Lao et al. (2021) demonstrating that EEAs facilitate effective resection of deep-seated orbital lesions with minimal complications. Moreover, advances in endoscopic technology and surgical expertise have broadened the applicability of EEAs, contributing to their growing preference among surgeons. While often labeled as “minimally invasive,” this characterization of EEA has been debated. The creation of a nasoseptal flap, wide bilateral sphenoidotomies, and extensive manipulation of sinonasal structures can result in non-negligible soft tissue trauma. This includes risks of postoperative nasal crusting, olfactory dysfunction, and septal perforation, which may not occur with the same frequency in some transcranial approaches. Thus, when weighing the advantages of EEA, it is crucial to also consider these potential morbidities (Chen et al., 2017). Despite these considerations, EEAs remain attractive for orbital cavernous malformation management due to their ability to provide direct access to medial orbital lesions, minimize brain retraction, and offer favorable cosmetic outcomes (Dallan et al., 2015a). When selected appropriately, they can still achieve high rates of gross total resection with fewer neurological complications compared to traditional open approaches.

Endoscopic transorbital approaches, despite their potential, remain underutilized, representing only a small fraction of cases in our review. While these approaches provide access to lateral and superior orbital lesions through eyebrow, eyelid, or conjunctival routes, their limited exposure and steep learning curve contribute to their restricted adoption (Agosti et al., 2021b, 2023; De Simone et al., 2024; Zoia et al., 2024c). Dallan et al. (2015b) highlighted the successful use of ETOAs in specific cases, yet their widespread application remains constrained. Nonetheless, with further refinement and increasing surgical expertise, ETOAs may become a more viable alternative in select cases (Zoia et al., 2019, 2023).

While it is conceptually accepted that surgical approach selection should be dictated by the anatomical location of the OCM, our analysis of the literature reveals a more complex and variable reality. Despite the theoretical alignment between lesion site and surgical corridor, several studies deviated from this paradigm. For instance, Boari et al. (2011) and Yang et al.(Kushwaha et al., 2024) reported resecting predominantly lateral lesions via midline transcranial approaches, such as the frontotemporal or pterional routes, rather than employing more direct lateral orbitotomies or transorbital techniques. Similarly, our review identified multiple cases where inferomedial lesions were accessed through extended transcranial exposures, despite the availability of endoscopic endonasal or transconjunctival options. This pattern suggests that, in many instances, surgical decision-making is influenced less by anatomical logic than by factors such as surgeon expertise, institutional familiarity with specific techniques, and access to specialized equipment (Missori et al., 1994; Maus and Goldman, 1999; Aymard et al., 2013; Kim et al., 2019). Moreover, we observed that centers contributing larger case series often adhered to a consistent approach across varied lesion locations, reinforcing the idea that personal or institutional preference can supersede lesion-specific tailoring. These findings emphasize a key limitation in the assumption that current practice reflects true anatomic customization.

Regardless of the surgical approach selected, the incorporation of image-guided navigation systems has become an essential adjunct in OCM surgery. While navigation offers value in both microsurgical and endoscopic procedures, its relevance is particularly pronounced in the context of endoscopic techniques, where conventional anatomical landmarks may be distorted, obscured, or absent due to prior interventions or lesion-related anatomical changes. In such scenarios, real-time navigation enables accurate localization and delineation of lesion boundaries relative to adjacent critical structures such as the optic nerve, medial rectus muscle, ophthalmic artery, and internal carotid artery (Kim et al., 2019). This facilitates safe dissection, minimizes the risk of iatrogenic injury, and improves the extent of resection. In endoscopic endonasal and transorbital procedures, where the field of view is limited and the working corridor is narrow, navigation compensates for these constraints by providing three-dimensional orientation and enhancing the surgeon's spatial awareness (Zoli et al., 2021). Moreover, navigation-guided surgery reduces reliance on extensive exposure, thereby preserving surrounding soft tissues and further supporting the minimally invasive paradigm. Studies have demonstrated that the use of intraoperative navigation can lead to decreased operative times, reduced complication rates, and improved surgical confidence, particularly during complex skull base or medial orbital interventions (Agosti et al., 2023).

### 4.3 Postoperative clinical and surgical outcomes

Resection rates remain a critical factor in evaluating surgical effectiveness. Several studies, including Aymard et al. (2013) and Calandriello et al. (2017), report high GTR rates using lateral orbitotomy and transcranial approaches, ranging between 85 % and 95 %. However, these techniques often require significant manipulation of the orbital contents, increasing the risk of optic nerve injury and prolonged recovery. In contrast, our systematic review aligns with the findings of Bleier et al. (2016a) and Castelnuovo et al. (2019), demonstrating that EEAs achieve comparable GTR rates (80 %–90 %) with minimal orbital disruption. This approach facilitates direct access to the medial orbital compartment via the skull base, avoiding unnecessary retraction and reducing surgical morbidity. Although EEAs may be less effective for laterally located OCMs, their success in medial lesions makes them a superior choice in many cases.

Postoperative complications further differentiate these approaches. Transcranial and lateral orbitotomy techniques, as reported by Dalfino et al. (2023) and de Melo Junior et al.(de et al., 2021), are associated with higher rates of periorbital hematoma (up to 20 %), optic nerve edema (10 %–15 %), and persistent diplopia (25 %–40 %). Our systematic review corroborates the findings of An et al. (2020) and Park et al. (2022), indicating that EEAs significantly reduce the risk of optic nerve injury and external scarring. However, EEAs have an inherent risk of cerebrospinal fluid (CSF) leaks (reported between 5 % and 15 %), necessitating meticulous skull base reconstruction techniques, such as nasoseptal flap coverage, to mitigate this complication. Notably, with advancements in endoscopic skull base reconstruction, CSF leak rates have substantially declined, reinforcing the safety profile of EEAs (Zoia et al., 2018).

Clinical outcomes and functional preservation are paramount in surgical decision-making (Fontanella et al., 2022; Clarós et al., 2019). Traditional transcranial and lateral orbitotomy techniques, despite achieving high resection rates, often lead to prolonged hospital stays and delayed recovery due to extensive tissue disruption. Additionally, patients undergoing EEAs report lower rates of postoperative diplopia and periorbital swelling, likely due to the minimally invasive nature of the procedure. Despite these benefits, EEAs may be limited in addressing laterally located lesions or those encasing major vascular structures, necessitating a tailored, case-specific approach.

### 4.4 Limitations

The primary limitation of this systematic review is the heterogeneity of the included studies, which complicates direct comparison due to variations in patient selection, lesion characteristics, surgical indications, and operative techniques. A significant proportion of the included literature (i.e., 56 out of 94 studies) consisted of case reports, which inherently limits the robustness of aggregated data. While these reports provide valuable anecdotal insights, they are subject to reporting bias and do not offer consistent or comprehensive data on complications or long-term outcomes. This aggregation of isolated experiences may therefore skew interpretation and dilute the reliability of conclusions regarding the comparative safety and efficacy of surgical approaches. Additionally, the expertise appears to be concentrated within a limited number of high-volume centers, with only 23 groups reporting more than 10 cases, highlighting a potential imbalance in experience distribution that may influence outcome generalizability.

The predominance of retrospective single-center studies and case series further reduces the overall level of evidence. Inconsistencies in reporting, particularly in relation to postoperative complications and long-term follow-up, limit the ability to assess recurrence rates or functional outcomes across surgical modalities. Moreover, data variability precluded meaningful statistical synthesis or meta-analysis. The lack of standardized outcome measures and reporting criteria across studies also increases the risk of publication bias, as favorable results are more likely to be published than studies with negative or inconclusive findings. Collectively, these limitations underscore the need for multicenter prospective studies with uniform outcome reporting to better define the optimal surgical strategy for orbital cavernous malformations.

Additionally, the analysis of postoperative complications was limited by inconsistent reporting across studies, particularly in distinguishing transient from permanent deficits. In many cases, complication details were sparsely described or variably categorized, limiting the ability to conduct a granular analysis. Importantly, complications are multifactorial events influenced by patient-specific factors, lesion size and location, surgical approach, and surgeon experience. Given these complex interdependencies, and the absence of individual patient data across studies, we were unable to perform a multivariate analysis to control for confounding variables. As such, while our findings suggest trends in complication rates associated with different approaches, these associations should be interpreted cautiously and not assumed to reflect direct causality.

Moreover, while a GTR rate of 94 % was reported across studies, the definition of resection completeness was often unclear or inconsistently applied. In many cases, it was not specified whether GTR was determined by intraoperative assessment or confirmed through postoperative imaging, limiting the objectivity and reproducibility of this outcome metric. Additionally, follow-up durations were generally short and heterogeneously reported. Only three studies included a follow-up period exceeding five years, which restricts our ability to assess the long-term behavior, recurrence potential, or delayed complications of orbital cavernous malformations. This limitation significantly impacts the interpretation of disease control and surgical durability over time.

## 5 Conclusions

Our systematic review highlights the predominancy of EEAs in the surgical management of OCMs, with an emerging role in the last years of ETOAs. Endoscopic techniques could offer a less invasive, direct route for both medially and laterally located lesions, reducing complications while preserving orbital function.

## Ethical approval

Not applicable.

## Funding

No funding was received for this research.

## Declaration of competing interest

The authors declare that they have no known competing financial interests or personal relationships that could have appeared to influence the work reported in this paper.

## Appendix A Supplementary data

Multimedia component 1 PRISMA checklist. Multimedia component 1

Multimedia component 2 NOS scores of each study included in the systematic review. Multimedia component 2
