# Supplementary material for: Surgical management and postoperative outcomes of orbital cavernous malformations: A systematic literature review by the EANS skull base section
Source: Brain Spine. 2025 Jun 22;5:104302. doi: 10.1016/j.bas.2025.104302 (PMC12268093; doi:10.1016/j.bas.2025.104302)
Supplement: Multimedia component 1 — PRISMA checklist. [file mmc1.docx]

| **Study** | **Year** | **Selection (4)** | **Comparability (2)** | **Outcome (3)** | **Total Score** | **Quality** |
| --- | --- | --- | --- | --- | --- | --- |
| Hobbs et al. | 1961 | 3 | 2 | 3 | 8 | High |
| Costa e Silva et al. | 1984 | 4 | 2 | 3 | 9 | High |
| Dyer et al. | 1985 | 3 | 2 | 2 | 7 | High |
| Lyness et al. | 1986 | 3 | 1 | 3 | 7 | High |
| Shields et al. | 1987 | 4 | 1 | 3 | 8 | High |
| Ohbayashi et al. | 1988 | 3 | 2 | 2 | 7 | High |
| McNab et al. | 1989 | 4 | 2 | 3 | 9 | High |
| Leatherbarrow et al. | 1989 | 3 | 1 | 3 | 7 | High |
| Leib et al. | 1993 | 3 | 2 | 2 | 7 | High |
| Missori et al. | 1994 | 4 | 1 | 3 | 8 | High |
| Hassler et al. | 1994 | 3 | 1 | 3 | 7 | High |
| Herman et al. | 1999 | 2 | 2 | 3 | 7 | High |
| Hejazi et al. | 1999 | 4 | 1 | 3 | 8 | High |
| Maus et al. | 1999 | 3 | 2 | 2 | 7 | High |
| D’hermies et al. | 2000 | 3 | 2 | 3 | 8 | High |
| Christensen et al. | 2002 | 4 | 1 | 2 | 7 | High |
| Kim et al. | 2002 | 3 | 2 | 3 | 8 | High |
| Schick et al. | 2003 | 4 | 1 | 3 | 8 | High |
| Papalkar et al. | 2005 | 3 | 2 | 2 | 7 | High |
| Monin et al. | 2005 | 3 | 1 | 3 | 7 | High |
| Karaki et al. | 2006 | 2 | 2 | 3 | 7 | High |
| Hejazi et al. | 2007 | 4 | 1 | 3 | 8 | High |
| Chaddad Neto et al. | 2007 | 3 | 2 | 3 | 8 | High |
| Maheshwari et al. | 2007 | 3 | 1 | 3 | 7 | High |
| Cheng et al. | 2008 | 4 | 2 | 3 | 9 | High |
| Yan et al. | 2008 | 3 | 1 | 3 | 7 | High |
| Stamm et al. | 2009 | 3 | 2 | 3 | 8 | High |
| Tang Chen et al. | 2010 | 4 | 2 | 3 | 9 | High |
| Yoshimura et al. | 2010 | 3 | 1 | 3 | 7 | High |
| Cho et al. | 2010 | 4 | 1 | 3 | 8 | High |
| Gazioglu et al. | 2011 | 3 | 2 | 2 | 7 | High |
| Campbell et al. | 2011 | 3 | 1 | 3 | 7 | High |
| Boari et al. | 2011 | 4 | 2 | 3 | 9 | High |
| Locatelli et al. | 2011 | 3 | 2 | 3 | 8 | High |
| Arora et al. | 2011 | 3 | 1 | 3 | 7 | High |
| Muscatello et al. | 2012 | 3 | 2 | 2 | 7 | High |
| Yamamoto et al. | 2012 | 3 | 2 | 3 | 8 | High |
| Meena et al. | 2012 | 3 | 2 | 2 | 7 | High |
| Netuka et al. | 2013 | 3 | 2 | 3 | 8 | High |
| Wu et al. | 2013 | 3 | 1 | 3 | 7 | High |
| Aymard et al. | 2013 | 4 | 2 | 3 | 9 | High |
| Murray et al. | 2013 | 3 | 1 | 3 | 7 | High |
| Healy et al. | 2014 | 3 | 2 | 3 | 8 | High |
| Chhabra et al. | 2014 | 3 | 2 | 2 | 7 | High |
| Yang et al. | 2014 | 4 | 1 | 3 | 8 | High |
| Dallan et al. | 2015 | 3 | 2 | 3 | 8 | High |
| Koch et al. | 2015 | 3 | 2 | 2 | 7 | High |
| Ikonomidis et al. | 2015 | 3 | 1 | 3 | 7 | High |
| Kang et al. | 2016 | 3 | 2 | 3 | 8 | High |
| Chen et al. | 2016 | 4 | 1 | 3 | 8 | High |
| Bleier et al. | 2016 | 4 | 2 | 3 | 9 | High |
| Xue et al. | 2016 | 3 | 2 | 2 | 7 | High |
| Wang et al. | 2017 | 3 | 1 | 3 | 7 | High |
| Louisraj et al. | 2017 | 3 | 2 | 3 | 8 | High |
| Bagheri et al. | 2018 | 4 | 2 | 3 | 9 | High |
| Bagheri et al. | 2018 | 3 | 2 | 3 | 8 | High |
| Golden et al. | 2018 | 3 | 1 | 3 | 7 | High |
| Dallan et al. | 2019 | 4 | 2 | 3 | 9 | High |
| Marcellino et al. | 2019 | 3 | 2 | 3 | 8 | High |
| Castelnuovo et al. | 2019 | 4 | 1 | 3 | 8 | High |
| Claros et al. | 2019 | 3 | 1 | 3 | 7 | High |
| Hegde et al. | 2019 | 3 | 2 | 2 | 7 | High |
| Kim et al. | 2019 | 3 | 1 | 3 | 7 | High |
| Zoli et al. | 2019 | 4 | 2 | 3 | 9 | High |
| An et al. | 2020 | 3 | 2 | 3 | 8 | High |
| Quin et al. | 2020 | 3 | 1 | 3 | 7 | High |
| Rimmer et al. | 2020 | 3 | 2 | 2 | 7 | High |
| May et al. | 2020 | 4 | 1 | 3 | 8 | High |
| Strianese et al. | 2021 | 4 | 2 | 3 | 9 | High |
| Fong Ng et al. | 2021 | 3 | 2 | 2 | 7 | High |
| De Feudis et al. | 2021 | 3 | 2 | 3 | 8 | High |
| Li et al. | 2021 | 4 | 2 | 3 | 9 | High |
| Almeida et al. | 2021 | 3 | 1 | 3 | 7 | High |
| Millesi et al. | 2021 | 3 | 2 | 3 | 8 | High |
| Lao et al. | 2021 | 3 | 1 | 3 | 7 | High |
| Zoli et al. | 2021 | 4 | 2 | 3 | 9 | High |
| Austria et al. | 2021 | 3 | 2 | 3 | 8 | High |
| Low et al. | 2021 | 3 | 2 | 2 | 7 | High |
| de Melo Junior et al. | 2021 | 4 | 1 | 3 | 8 | High |
| Park et al. | 2022 | 3 | 1 | 3 | 7 | High |
| Zhou et al. | 2022 | 3 | 2 | 3 | 8 | High |
| Essayed et al. | 2022 | 3 | 2 | 2 | 7 | High |
| Yang et al. | 2022 | 4 | 1 | 3 | 8 | High |
| Ayoub et al. | 2022 | 3 | 2 | 3 | 8 | High |
| Almatrudi et al. | 2023 | 3 | 2 | 2 | 7 | High |
| Lai et al. | 2023 | 3 | 1 | 3 | 7 | High |
| Leocata et al. | 2023 | 3 | 2 | 3 | 8 | High |
| Jaxa-Kwiatkowski et al. | 2023 | 3 | 2 | 3 | 8 | High |
| Finisanti et al. | 2023 | 4 | 1 | 3 | 8 | High |
| Das et al. | 2023 | 3 | 2 | 2 | 7 | High |
| Dalfino et al. | 2023 | 4 | 2 | 3 | 9 | High |
| Gulsuna et al. | 2024 | 3 | 1 | 3 | 7 | High |
| Kushwaha et al. | 2024 | 3 | 2 | 3 | 8 | High |
| Abdulla et al. | 2024 | 4 | 1 | 3 | 8 | High |
